# Novel Cell-Based and Tissue Engineering Approaches for Induction of Angiogenesis as an Alternative Therapy for Diabetic Retinopathy

**DOI:** 10.3390/ijms21103496

**Published:** 2020-05-15

**Authors:** Elmira Jalilian, Kenneth Elkin, Su Ryon Shin

**Affiliations:** 1 UCL Institute of Ophthalmology, University College London, London EC1V 9EL, UK; 2 Wayne State University School of Medicine, Detroit, MI 48201, USA; kenneth.elkin@med.wayne.edu; 3 Division of Engineering in Medicine, Department of Medicine, Harvard Medical School, Brigham and Women’s Hospital, Cambridge, MA 02139, USA; sshin4@bwh.harvard.edu

**Keywords:** diabetic retinopathy, ischemia, stem cell, vascular regeneration, vascular tissue engineering

## Abstract

Diabetic retinopathy (DR) is the most frequent microvascular complication of long-term diabetes and the most common cause of blindness, increasing morbidity in the working-age population. The most effective therapies for these complications include laser photocoagulation and anti-vascular endothelial growth factor (VEGF) intravitreal injections. However, laser and anti-VEGF drugs are untenable as a final solution as they fail to address the underlying neurovascular degeneration and ischemia. Regenerative medicine may be a more promising approach, aimed at the repair of blood vessels and reversal of retinal ischemia. Stem cell therapy has introduced a novel way to reverse the underlying ischemia present in microvascular complications in diseases such as diabetes. The present review discusses current treatments, their side effects, and novel cell-based and tissue engineering approaches as a potential alternative therapeutic approach.

## 1. Introduction

Diabetes mellitus is a chronic metabolic disease characterised by sustained hyperglycemia that leads to macro and microvascular complications [1]. Diabetes is the leading cause of blindness among adults aged between 20 and 79 years old. Recent surveys have predicted that by 2030, the number of patients with diabetes mellitus will increase to 440 million worldwide (prevalence 7.7%) [1]. Globally, diabetes will lead to increasing incidence of two major types of late complications: macrovascular and microvascular, which cause greater morbidity and premature death. Cerebrovascular, cardiovascular and peripheral vascular diseases are examples of macrovascular disorders in which large vessels are affected. In contrast, microvascular complications affect small vessels and include nephropathy, neuropathy, and retinopathy. Retinopathy is one of the most common ischaemic disorders of the retina and the main cause of blindness in the working-age population. It is responsible for 12,000–24,000 new cases of blindness each year worldwide [2,3,4]. 

Diabetic retinopathy (DR) manifests as a broad spectrum, particularly at the level of the retinal vasculature, and is responsible for 4.8% of the 37 million cases of blindness in the world according to the World Health Organization (WHO). The main risk factors for DR are high blood pressure, hyperglycemia, and the duration of diabetes. Studies have found consensus that there is a pathogenic link between hyperglycemia and the onset and progression of DR, while tight control of blood glucose can delay DR onset and progression. Some of the DR risk factors are gender, age at onset of the disease, ethnicity, cataract extraction, and hyperlipidemia [2]. The duration of diabetes is another main risk factor for DR. Although type 1 and type 2 diabetes have some different phenotypic variations, the prevalence of diabetic retinopathy in both populations after 10 years is approximately 75% which increases to 90–95% after 20 years. Despite the increasing number of diabetic patients during the last decade, most of therapeutic applications only result in reducing the pathogenic process and not affecting the underlying cause of the DR. Therefore, there is an urgent need to investigate novel approaches to address the problem. In this review, we first explain the pathogenesis of DR and current therapeutic approaches, and then will discuss novel cell base and tissue engineering approaches. Tissue engineering strategies have three basic components: first, the cell source which must express the appropriate genes and maintain the appropriate phenotype in order to preserve the specific function of the tissue [5]. Second, the bio-reactive agents or signals that induce cells to function. third, the scaffolds that house the cells and act as a substitute for the damaged tissue [6]. The source may be either embryonic stem cells (ESC) or adult stem cells (ASC), the scaffolds may be categorised as synthetic, biological, or composite, and the signals may include growth factors/cytokines, adhesion factors, and bioreactors [5]. 

### 1.1. Vascular Insufficiency and Inner Retinal Ischemia in Diabetic Retinopathy

Ischemia is characterised by the restriction of blood supply to tissue and organs, causing a shortage of oxygen and glucose which is needed for cellular metabolism and removal of metabolites [3]. Ischemia-related pathologies are central to many diseases and pose a challenge for healthcare systems worldwide. Angina, myocardial infarction, stroke, and ischaemic retinopathies are some of the most common ischemia-related diseases which represent a major cause of morbidity and mortality worldwide [6].

Vaso-degenerative retinopathies, such as DR, can result in variable degrees of retinal vascular insufficiency and a profound loss of vision. Beyond the significant risk of depriving delicate neural networks of oxygen and nutrients, hypoxia also increases growth factor and cytokine expression. This can result in vascular leakage in the surviving vasculature and/or pre-retinal and papillary neovascularization. If these complications are left untreated, the responses to vascular stasis, ischemia or hypoxia can result in fibro-vascular scar formation or retinal edema and blindness [3,7].

### 1.2. Clinical Signs and Diagnosis

Many diabetic patients may not experience any noticeable symptoms in the early stage of the disease. However, early detection of DR can help to prevent severe loss of vision and blindness. Different clinical signs of retinopathy include dot and blot retinal hemorrhage, the formation of microaneurysms, cotton wool spots, hard exudates, venous abnormalities, and growth of new blood vessels. There are also anatomical changes during DR that have been well-documented and include the formation of acellular capillaries, early thickening of the basement membrane, formation of microaneurysms, loss of pericytes and endothelial cells, and retinal neovascularization [8]. DR diagnosis involves visual acuity testing, fundus examination (direct and indirect ophthalmoscopy) and retinal photography. Optical coherence tomography (OCT) is widely used to examine the major layers of the retina and the various reflectance of visible light [9,10]. By using this technique, it is possible to localize retinal lesions in relation to different retinal layers and to quantify the retinal thickness. Furthermore, OCT is also used to measure retinal blood flow and diagnose retinal edema [4]. 

### 1.3. Classification and Treatments 

**Figure 1:**
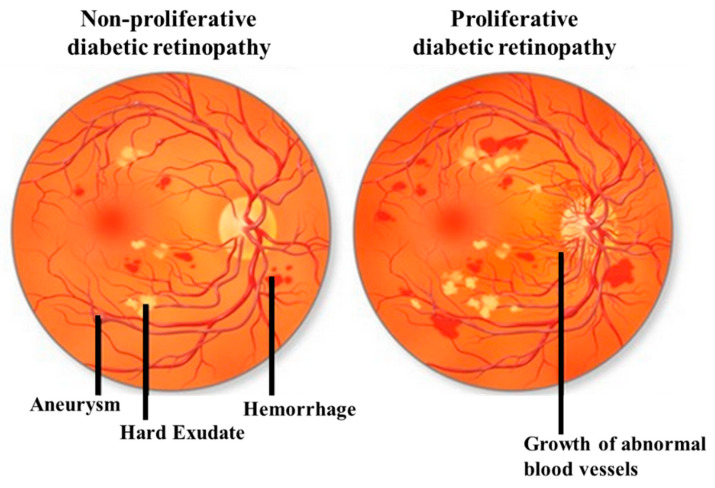
Illustrative pictures of non-proliferative diabetic retinopathy (DR) (NPDR) (left) and proliferative DR (PDR) (right). In early-stage NPDR, damaged blood vessels begin to leak extra fluid and blood into the eye. In PDR, many retinal blood vessels are closed, disrupting blood flow. In response to hypoxia, new blood vessels are generated (neovascularization), which are abnormal and ineffective. Illustration with permission from https://maxivisioneyehospital.wordpress.com.

DR can be classified by the clinical presentation either as non-proliferative DR (NPDR) or as proliferative DR (PDR). The first change observed in DR patients is a reduction in the retinal blood flow, which is followed by a loss of pericytes resulting in the development of micro-aneurysms, which may be associated with the appearance of retinal hemorrhages and hard exudates (Figure 1). These changes are collectively referred to as NPDR. Basement membrane thickening and leakage results in the first noticeable abnormality of NDPR. As the vascular damage progresses and a wider area of ischemia develops, neovascularization may become evident in the retina and over the optic nerve. Vascular endothelial growth factor (VEGF) is then released to develop a new nutrient supply by constructing capillary tubes. This is the stage where DR becomes PDR. These new blood vessels are fragile and tend to bleed, and cause scarring on the surface of the retina. This is the most advanced and serious form of diabetic retinopathy [11].

At any stage of the disease, DR can be associated with diabetic macular edema (DME), DME is defined as retinal thickening caused by vascular leakage and a build-up of fluid and proteins within two-disc diameters of the macular region. DME is the major cause of severe visual impairment in diabetic patients. Diabetic macular ischemia (DMI) occurs when small blood vessels close completely over time, resulting in poor blood flow. DMI causes the death of nerve cells in the macula responsible for fine vision, which is an irreversible process resulting in a permanent, untreatable central blind spot which decreases central vision [12].

NPDR and DME are considered the most sight-threatening ocular complication. Many studies have demonstrated that prevention and modification of associated systemic risk factors are the critical steps for the treatment of diabetic retinopathy. The control of blood pressure, blood glucose and glycosylated hemoglobin levels, and lipid levels have all been associated with the reduction of the long-term risk of developing sight-threatening ocular complications. Much research has been carried out worldwide and has led to various novel therapeutic targets. The Early Treatment Diabetic Retinopathy Study (ETDRS) established pan-retinal and macular laser as the gold standard treatment for these complications [11]. 

Laser photocoagulation and vitreoretinal surgery (vitrectomy) are the current surgical therapies that are effective in reducing the loss of vision and are useful for the late-stage disease of retinopathy but carry significant sight-threatening side effects [13]. Although laser photocoagulation and pars plana vitrectomy have been shown to be useful in the treatment of severe visual loss in DR patients, visual loss continues after therapy [14]. Recently, the discovery of vascular endothelial growth factor (VEGF) and its important angiogenic role has sparked new, potential therapeutic approaches. Clinical studies have demonstrated the role of VEGF in the pathogenesis of DME and exudative AMD [15,16,17]. Damage to the retinal microvasculature results in elevated intraocular levels of VEGF, a pathophysiologic mediator in PDR and DME [18]. VEGF is also associated with breakdown of the blood-retina barrier, causing increased vascular permeability which results in vascular edema. High levels of VEGF, in a different study, were found in ocular fluids of patients with PDR and DME [19]. This has led to the application of anti-VEGF drugs to treat PDR and DME in combination with existing therapies. Currently, four VEGF-binding drugs including Pegaptanib, Ranibizumab, Bevacizumab, and Aflibercept (Table 1) have received U.S. Food and Drug Administration (FDA) approval for different diseases and are currently in trial for the treatment of diabetic retinopathy.

It is important to mention that there are also other vascular mediators that has been shown to contribute to ocular angiogenesis including members of platelet-derived growth factor family (PDGF), fibroblast growth factor family (FGF), transforming growth factor-β superfamily (TGF-β1), epidermal growth factor family (EGF), hypoxia-inducible factors insulin-like growth factors, cytokines, matrix metalloproteinases and their inhibitors, and glycosylation proteins. Recent studies investigated new drugs such as Fovista (PDGF), X-82 (VEGF & PDGF), Squalamine lactate (bFGF, PDGF& VEGF-A), and RG7716 (ANG-2 and VEGF) to target retinal neovascularization for future anti-angiogenic therapies. Full description of ongoing clinical trials of new drugs against retinal and choroidal angiogenesis are shown in Table 1 of Cabral et al. [20]. Although regulating these networks of factors has recently shown to be more effective than focusing on any single one like VEGF, novel molecules should be investigated further as targets for future anti-angiogenic therapies.

Despite promising results with anti-VEGF therapy, some important issues should be considered. First, the requirement of multiple intravitreal injections can cause side effects, including cataracts, uveitis and retinal detachment [21]. Furthermore, it has been reported that some patients with DR respond poorly to VEGF inhibition and, in some cases, is associated with a poor visual outcome [22,23]. Second, current therapies are only applicable for proliferative disease and DME and aim to mitigate the results of the pathogenic process without affecting the underlying cause. Third, in a subgroup of patients with pure DMI, where small blood vessels close off completely and the retina slowly degenerates, there is absolutely no indication of using anti-VEGF drugs. VEGF has additionally been shown to influence neuronal growth and differentiation [24], and to reduce the number of apoptotic retinal cells in response to ischemia, which was shown to be reversed after adding a VEGF inhibitor [25]. Therefore, using anti-VEGF therapy to inhibit unwanted angiogenesis might inadvertently inhibit adult neurogenesis and neuroprotection [26]. These current treatment approaches do not address the primary problems during the early stage of the disease. In these instances, regenerative medicine might introduce an alternative way to regenerate areas of vaso-degeneration and may reverse ischemia by regenerating blood vessels. 

Vascularisation is one of the most critical challenges to be addressed in tissue engineering so it can be clinically implemented. There are several important criteria that should be considered to achieve sufficient tissue-engineered vasculature. Cells should be located an optimum distance between a patterned vascular network and the surrounding tissue for proper oxygen and nutrient exchange. Vascular lumen must line up with the endothelium for adequate homeostasis and material exchange to enable quick integration with surrounding host tissue and development of a long-lasting vascular network [27].

Within the vasculature hierarchy, the size of blood vessels can be significantly different which varies the design requirements and approaches for bioengineering [28]. Small vessels like arterioles and capillaries have diameters in the sub-millimeter scale (>6 mm in diameter), and thus present a different set of challenge. Against clinical need for large vessels which is to fabricate a single tube of smaller vessels, in microvessels fabrication a network of nutritive (high endothelium surface-area-to-volume ratio) is needed. To this end, hydrogels or scaffolds are used to provide regenerative cues to induce the formation of a network of nutritive vascular bed and retain the vascular network at the target implant site [29]. Therefore, in this review after briefly explaining the blood vessel regeneration during the embryonic development, since diabetic retinopathy is one of the ischemic conditions arising from microvasculature insufficiencies, we will next explore recent advances in fabricating blood vessels that comprised of microvessels and will discuss the current methods used in tissue engineering and cell therapy approaches to generate microvessels and how they facilitate the integration of implanted vasculature within a host. 

## 2. Development of the Vascular System during Embryogenesis 

After fertilization, the zygote divides mitotically and generates a morula (termed blastula in invertebrates). After the first series of cell divisions, the blastula/morula undergoes a spatial reorganization called gastrulation. This process starts with an infolding (primitive streak) of the single-layered blastula, eventually leading to three distinct germ layers known as the ectoderm (outer layer which produces cells of the epidermis and nervous systems), endoderm (inner layer which produces most of the internal organs), and mesoderm (middle layer which gives rise to muscles, the heart, the vasculature, and bone) [30]. The circulatory system is one of the lateral mesodermal derivatives appearing in the third week of embryonic development in humans. The development starts with the formation of blood islets in the yolk sac and hemangioblasts (bipotent cells giving rise to both hematopoietic and angioblastic cells) in the head mesenchyme and posterior-lateral plate mesoderm. The emergence of scattered of precursors of endothelial cells (so-called angioblasts) through the mesoderm results in the formation of clumps, and then cords, which consequently differentiate into endothelial cells and functional vessels [31].

**Figure 2:**
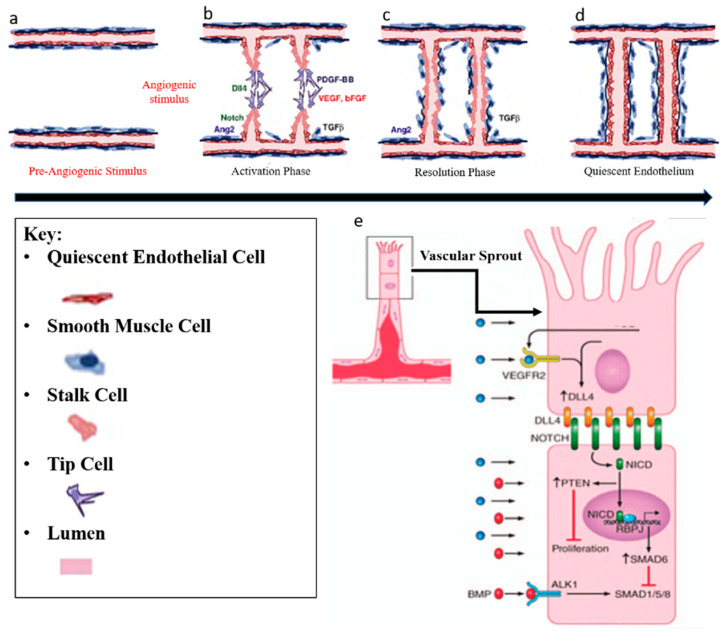
Angiogenesis regulation. Angiogenesis includes two phases. (**a**) In the activation phase, the basement membrane is degraded by angiogenic stimulus (VEGF, bFGF, and TGF-ß) (**b**) and tip cells invade the tissue by extending filopodia. Stalk cells proliferate and extend, and new branches join in tip-cell-tip-cell fusion. (**c**) Lastly, in the resolution phase, endothelial cells (ECs) stop proliferating and mature by re-formation of basement membrane (**d**) and obtain a quiescent phenotype which is called phalanx EC. Illustration with permission from [37]. (**e**) VEGF signalling induces Dll4 expression in tip cells and consecutively, Dll4 activates Notch signalling in stalk cells. This results in reducing stalk-cell sensitivity to VEGF stimulation, which in turn suppresses the tip cell phenotype. Illustration with permission from [38,39].

Blood vessels develop by a combination of vasculogenesis and angiogenesis. Vasculogenesis describes the de novo formation of blood vessels and is responsible for the formation of primary vessels during early embryonic development such as the dorsal aorta. It relies on the local differentiation of mesoderm-derived precursors of endothelial cells (PECs) into ECs that coalesce into primitive networks. Angiogenesis is the expansion of a pre-existing vessel network through a combination of sprouting, proliferation, and remodeling processes [32]. In adult life, angiogenesis occurs only during inflammation, wound healing, the female menstrual cycle, and in numerous pathological disorders including retinopathies, rheumatoid arthritis and tumor growth [33]. Angiogenesis includes two phases. In the activation phase, the basement membrane is degraded (Figure 2a) by the angiogenic stimulus and VEGF signaling induces DLL4 expression in tip cells in the front position of sprouting vessels invade the tissue by extending filopodia (Figure 2b). DLL4 activates Notch signaling in Stalk cells (Figure 2e) which results in proliferation and extension of Stalk cells, and the new branches join over tip-cell-tip-cell fusion (Figure 2c). Second, in the resolution phase, ECs stop proliferation and mature by re-formation of basement membrane and obtain a quiescent phenotype, which is called phalanx EC (Figure 2d) [34]. The specification of tip and stalk cell identities is a dynamic process and interplay between VEGF and Notch signalling pathways has shown to be responsible during this process [35]. All these EC phenotypes have their specific gene transcriptional profile. Both, the proliferative stalk cells and the quiescent phalanx cells are covered by smooth muscle cells and pericytes (so-called “mural cells”). Mural and endothelial cell interactions play an important role in vessel maturation and differentiation, indicating great potential utility in vascular tissue engineering [36].

## 3. Cell Therapy-Based Approach for Ischemic Disease 

To date, many cell-based approaches have used vascular endothelial cells as a potential source to regenerate blood vessels because of their ability to self-assemble into functional capillary network and integrate into host tissue. However, several concerns emerged. For instance, terminally differentiated ECs, such as microvascular endothelial cells, are limited in expansion potentials and it has been shown that isolation and purification of these populations need further invasive procedures. Additionally, not all ECs have the same functional properties due to the donor variability and tissue site septicity. Several in vitro and in vivo studies exhibited better functional phenotypes, higher vascular density, and more stable blood vessels when ECs co-cultured with other cell types such as pericytes, fibroblasts or MSCs compared to ECs alone [40,41,42]. Therefore, due to various limitations of ECs, shift of attention has been implicated to other cell sources including induced pluripotent stem cells (iPSCs), embryonic stem cells (ESCs), or different types of adult stem cells such as endothelial progenitor cells (EPCs) [43], umbilical vein endothelial cells (HUVECs), or mesenchymal stem cells (MSCs). These cells have been shown to grow fast in in vitro conditions with high repopulating potentials, demonstrate angiogenic properties, and, thus, are great sources for clinical applications. 

### 3.1. Endothelial Progenitor Cells (EPC)

Progenitors of endothelial cells are known to be a promising stem cell source for vascular regeneration [44,45]. They are typically derived from adult stem cells including peripheral blood (PB) [46], cord blood (CB) [47], and bone marrow (BM) [48]. However, controversy over the origin, differentiation and cellular identity of these cells remains a potential issue. Using adult stem cells to regenerate blood vessels was first introduced by Asahara in 1997. It was believed that postnatal neovascularization was exclusively based on fully differentiated ECs, derived from pre-existing blood vessels. However, Asahara showed that putative hematopoietic precursors cells (CD34+, Flk-1+/KDR+) from human adult circulating blood cells can differentiate to ECs in vitro and called them “endothelial progenitor cells (EPCs)” [49]. To date, there are several markers used to identify these cells [50,51]. However, there is considerable debate about the true nature of EPCs and the specificity of these markers [52,53]. CD34 is the main marker of these cells. CD34 + mononuclear blood cells after seven days of in vitro culture were observed to lose the expression of leukocyte marker CD45 and differentiated to EC-like phenotype expressing all EC lineage markers CD31, KDR, Tie2, and E-selectin [54]. More interestingly, injection of CD34+ cells into the ischemic hindlimbs of a mouse model showed incorporation of these cells into the site of active angiogenesis and contribution to the tissue vascularization [55,56,57]. With regard to diabetic retinopathy, endothelial progenitor cell (EPC) therapy has represented an exciting alternative strategy to the current end-stage approaches for this disease, with animal studies showing promoted vascular repair in the retina of a mouse model of oxygen-induced retinopathy [58]. These cells were shown to differentiate to microglia [59] and significantly improved vascular regeneration [60,61]. However, preclinical evidence for the ability of EPCs to stimulate vascular regeneration is controversial, and some studies have failed to demonstrate any beneficial outcome from stem cell therapy [52,62]. EPCs from diabetic patients with vascular complications are dysfunctional, posing its own challenges and complicating the use of autologous EPCs [58]. The variation of EPC therapy may be also due to differences in the target tissues studied genetic variability in the animal strains and, perhaps most importantly, due to the heterogeneity of EPCs based on their isolation and culture methods [63].

**Figure 3:**
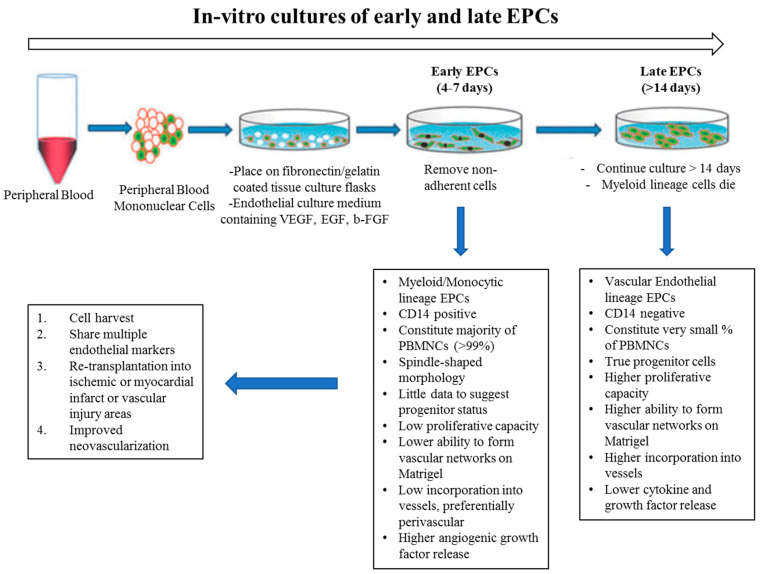
In vitro culture of endothelial progenitor cells (EPCs); early vs late outgrowth. EPCs are grown from whole peripheral blood mononuclear cells. Early EPCs are obtained from short term culturing (4–7 days) on fibronectin. A small population of EPCs when plated for >14 days is called late-outgrowth EPC (or ECFC) and demonstrate an increased capacity for proliferation and blood vessel formation. Illustration with permission from [64].

In vitro studies have suggested that there are at least two different types of EPCs: (1) early and (2) late outgrowth EPCs. Although both of these populations showed the capacity to promote vessel regeneration in different animal models, they illustrated different capabilities to differentiate into ECs and to physically contribute to new blood vessel formation. “Early EPCs”, also called non-colony forming EPCs, have myeloid/hematopoietic characteristics and share lineage traits with immune cells, specifically macrophages and monocytes. They are isolated from adult peripheral blood mononuclear cells (PB-MNCs) or human cord blood mononuclear cells (CB-MNCs), which are plated on Fibronectin-coated dishes for 48hrs to deplete the adherent macrophages and mature ECs. Then, non-adherent cells are removed and re-plated on Fibronectin-coated plates and VEGF containing medium. After 4–7 days, so-called “early EPCs” can be obtained (Figure 3). These cells secrete pro-angiogenic factors and are likely related to what is known as circulating angiogenic cells (CACs) [64]. They have myeloid characteristics, typically not forming colonies under conventional endothelial differentiation conditions [65]. They are suggested to have paracrine effects in angiogenesis by secreting factors, rather than in integrating into the endothelium. In contrast, when collagen-coated plates are used, after 2–4 weeks of culturing, late outgrowth EPCs (OECs) emerge (Figure 3). These cells have been shown to exhibit all of the typical markers and functional characteristics of mature endothelial cells [66,67]. Other groups refer to these cells as endothelial colony-forming cells (ECFCs) [68]. They have a cobblestone appearance, high proliferation capacity, can differentiate to EC, and they have been observed to physically contribute to new vessel formation in vitro and in vivo. Considering the diverse characteristics of EPCs, and to achieve the ideal “cell therapy,” approach, the optimal EPC must be determined, any functional dysfunction must be corrected prior to use, and the diabetic milieu will require modification to accept the EPCs.

With regards to in vitro studies, EPCs have shown to generate lumenized capillary-like structures on Matrigel and a partially functional capillary network inside a modified poly (ethylene glycol) (PEG) hydrogel in the presence of mural cells [69]. The importance of co-culture system in final elevated vascularisation was confirmed in different studies. Blood-derived EPCs co-cultured with MSCs (ratio of 1:1) and suspended in Matrigel illustrated numerous luminized networks when injected into the nude mice [70]. EPCs have also been successfully used to cellularize biodegradable vascular grafts for endothelialization and in vitro maturation [71]. Taken together, these pioneering studies validate the utility of EPCs as a promising cell source to pre-vascularize tissue-engineered grafts. It is important to emphasize that having an ideal cell source is not sufficient for therapeutic efficacy of delivered cells. Therefore, recent tissue engineering efforts have appropriately focused on recreating the architecture and function of the vasculature in vitro prior to implantation, with the hypothesis that pre-vascularized grafts and tissues enhance integration with the host. 

### 3.2. Pluripotent Stem Cells in Vascular Regeneration

**Figure 4:**
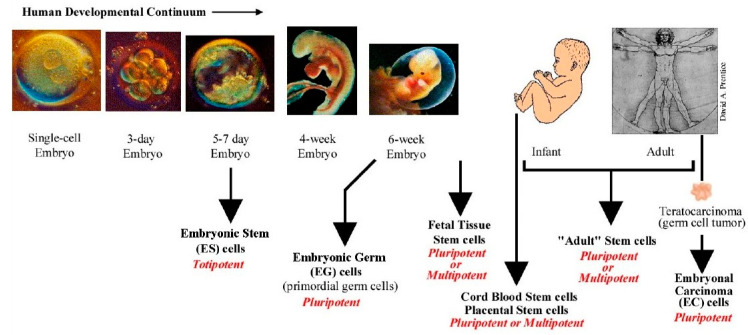
Various sources of stem cells. ES cells are isolated from the inner cell mass (ICM) of the blastocyst and are considered pluripotent. Primordial germ cells are derived from embryonic germ cells and are pluripotent. Fetal stem cells are derived from the developing foetus and are pluripotent or multipotent. All stem cells derived after birth are adult stem cells (ADS). These cells have limited potential and are usually multipotent. Cord blood derived stem cells occupy a niche between ES cell and ADS. These have been categorized to be pluripotent and display some ES cell-like properties. Illustration from www.stemcellresearch.org/testimony/images/20040929prentice.htm.

Stem cells may be sourced from blastocysts before implantation from day 5–7 of the embryo (embryonic stem cells, or after six weeks from foetus, which are considered less pluripotent stem cells (foetal stem cells). Other types of stem cells can be derived from blood or other tissues postnatally (adult stem cells), as seen in (Figure 4). [72] Each of these stem cells play a unique role in stem cell research and therapeutic applications [73]. More recently, stem cells can also be generated from somatic cells by re-programming strategies and are called induced pluripotent stem cells (iPSCs) [74].

### 3.3. Embryonic Stem Cells in Vascular Regeneration

Researchers have successfully derived ECs and SMCs from human embryonic stem cells (hESC) lines. Several different approaches have been used to differentiate SMCs from hESCs. Endothelial progenitor cells such as CD34+ cells have been isolated either from spontaneous differentiation of embryoid bodies (EBs) or from co-cultures and then induction with factors such as TGF-β1, PDGF-BB, and retinoic acid [75,76]. Other groups have investigated 2D and 3D culture systems to derive hES-derived ECs. These studies illustrate that these cells form vessel-like structures on Matrigel, and that they integrate into the host tissue and form blood vessels that were functional for 150 days when were transplanted into SCID mice [5]. More details about 2D and 3D assays discussed in further paragraphs.

### 3.4. Induced Pluripotent Stem Cells in Vascular Regeneration 

iPSC cells are a type of PSCs that can be prepared by re-programming adult somatic cells into stem cells, initially illustrated by Yamanaka in 2006 [77]. iPSC technology allows us to derive patient-specific cells, which avoids some of the ethical concerns surrounding ESCs, allograft rejection, immunogenicity, and also allows us to scale up production of the desired cell lineage and generate new prospects for regenerative medicine [78,79]. Several concerns, including ethical issues, immunological barriers, high regenerative potentials and unintentional proliferation of ESCs, can be overcome by using iPSCs [80,81]. iPSCs are easily accessible from different sources such as skin, hair or blood, and there are no ethical concerns surrounding these cells [82,83]. Studies on iPSC cells have shown their potential to differentiate into all cardiovascular compartments, such as pericytes, smooth muscle cells, Ecs, and cardiomyocytes [84,85]. 

It has been shown that iPSC-derived Ecs are able to incorporate into damaged vasculature of ischemic tissue and improve function [86,87]. However, several concerns remain regarding the use of iPSCs in vascular regeneration. Markers and reproducible protocols are required to differentiate iPSC into the vascular lineage. As with ESCs, it is crucial to efficiently exclude pluripotent cells to avoid teratoma formation [81]. However, the main concern about using iPSCs is the clinical development of these cells. Genetic manipulation of iPSCs using retrovirus or lentivirus results in the integration of viral DNA into the chromosome, increasing the risk of silencing indispensable genes or inducing ontogenesis [88]. Although using adenoviruses or plasmids has reduced these risks in part, even episomal vectors carry risks of DNA integration [89]. Another concern in using iPSCs is retaining the presence of genetic and acquired abnormalities from the patients’ cells and transferring them into their iPSC cells, which reduces their regenerative capacity and contributes to vascular inflammation. Even when considering all advantages and disadvantages of embryonic versus adult stem cells, there still exist several controversies about their feasibility for use in blood vessel regeneration. Recent gene transcriptional profiling has shown that iPS-derived progenitors of endothelial cells (PECs) are fundamentally different from adult derived endothelial progenitor cells (EPCs), a difference which must be considered when these cells are used in clinical applications [90]. Due to the fact that iPS-derived PECs are “true progenitors” and due to the diversity of the sources of adult stem cells (bone marrow, peripheral blood and cord blood), it is anticipated that using iPS/embryonic stem cells may generate more defined endothelial cells with enhanced morphology and phenotype for tissue engineering applications [91]. 

### 3.5. Human Mesenchymal Stem Cell (MSCs) in Vascular Regeneration

Human adult MSCs, identified in different tissues such as bone marrow and adipose tissues, are a type of adult multipotent stem cell with a more restricted differentiation potential and limited self-renewal ability [92]. Studies have illustrated the potential of these cells to differentiate into ECs after seven days of exposure to VEGF and bFGF, and expressing different EC markers such as Flk-1, vWF, and VE-cadherin. Moreover, human adipose-derived MSCs plated in endothelial growth medium-2 (EGM-2) containing VEGF form vessel-like structures on Matrigel and expressed ECs markers CD31, vWF, and eNOS. The potential of EC-derived MSCs has been further investigated in in vivo studies, and have been shown to improve muscular angiogenesis and restoration of blood perfusion in a mouse hindlimb ischemia model [93,94]. MSCs have been used as supportive cells to maintain the functionality of engineered vasculature through various mechanisms, and are illustrated to mediate angiogenesis by secreting of pro-angiogenic factors including HGF, IGF, bFGF, and VEGF which improve EPC differentiation into ECs [70]. MSCs can also differentiate into vascular smooth muscle cells through TGF-beta induction, and can express smooth muscle actin (SMA) and H1-calponin [95]. It has shown that EPC implants are capable to form human micro-vessels in mouse models only in the presence of MSCs [70]. However, the concerns about MSC implementation in clinical application is their heterogenic nature and their high individual variability. Overcoming these obstacles is critical in the field of MSC-derived vascular tissue engineering so that vascularisation outcome can be optimised [96]. 

## 4. Vascular Tissue Engineering Technology

There are several 2D and 3D assays to develop tube-like structures. In 2D wound-healing assay, cells are cultured in a monolayer and, when cells are confluent, a “wound” is created and images captured from the beginning and at specific intervals during cell migration until the wound is closed. This method was one of the very early methods to study the directional cell migration in vitro [97]. More recently, the 3D aortic ring assay has been developed that is based on organ culture. In this assay, rat thoracic aorta is excised, the fat layer and adventitia removed, and small ring-like segments approximately 1mm in size are cut and embedded into a three-dimensional matrix composed of fibrin or collagen. They are maintained in a chemically defined medium, where angiogenic factors and inhibitors of angiogenesis can be directly added to the rings. In this way, new blood vessel growth and paracrine angiogenic effects can be investigated. Therefore, the aortic ring assay is a more physiologically relevant assay to study sprouting angiogenesis responses [98,99]. The in-vitro collagen lumen assay is another 3D assay used to differentiate endothelial cells in 3D microenvironment. In this method, ECs are plated in two layers of collagen. Soon after it is formed, cells undergo morphogenesis to form a structure similar to the capillary network and appropriately polarized luminal structures [100,101] A third type of 3D angiogenesis assay, called a “fibrin gel bead assay”, endothelial cells are coated onto cytodex microcarriers and embedded into a fibrin gel. To provide suitable factors, fibroblasts are cultured on top of the gel where they secrete factors and promote endothelial cell sprouting from the surface of the beads. After several days, many vessels are presented and can be observed under phase-contrast microscopy [102]. Additionally, the retina explant assay is one of the more powerful assays for the retina. The assay involves plating a small piece of retina on an organotypic filter. In this assay, retina explants can be prepared anytime between embryonic day 13 and postnatal day 4, from which point the explants will develop very similarly to a retina in vivo and generate all of the different retinal cell types that will migrate to the appropriate layer. This assay has been shown to be very useful in the study gene function at different embryonic stages [103,104]. All of these studies and assays illustrate that endothelial cells form vessel-like structures on Matrigel, and that they can integrate into the host tissue, forming blood vessels that were functional for 150 days when transplanted into severe combined immunodeficient mice (SCID) mice [5]. Ideally, tissue-engineered vasculature that is designed for therapy must be transplantable or must stimulate vasculature formation at the transplant site. Further, engineered vessels for implantation must withstand physiological pressures without leakage or aneurysm formation, should not be thrombogenic, and should not elicit an immunological response from the patient. For clinical applications, the time that a patient must wait for a vascular therapy should be consistent with the clinical indication for use. Blood vessels range in size and include microvessels (<1 mm), small vessels (1–6 mm), and large vessels (>6 mm in diameter). Blood vessels for inducing the angiogenesis in diabetic retinopathy is categorized in microvessels, hence the focus on tissue engineering approaches in micro-scale. These approaches include stimulation of angiogenesis in vivo, by implantation of endothelial cells, or by re-endothelialization of decellularized organs. In addition, micro- fabrication technologies have recently developed as a promising approach for the future of in vivo vascular tissue engineering [105].

### 4.1. In Vivo Microvascular Network Formation Using Pro-Angiogenic Stimuli 

Several studies have shown that angiogenic stimulation can occur in vivo by using a variety of cytokines and gene-based and cell-based approaches. Pre-clinical studies, for instance, have shown successful angiogenic response in a rabbit hind limb ischemia model to form collateral vessel formation by intra-arterial injection of VEGF [106,107,108,109]. However, it should be mentioned that the same studies in human patients have not been as promising, potentially as a result of low progenitor cells with low circulatory efficiency and impaired inflow in elderly human patients. Therefore, it has been suggested that angiogenic stimulation therapy should begin much earlier to augment therapeutic efficacy. Furthermore, angiogenesis is a complex and high regulated process that may require a multidisciplinary approach incorporating chemistry, material science, and biology in addition to cytokine-based angiogenic stimulation [105].

### 4.2. Implantable Engineered Micro-Vascularised Tissue 

Endothelial cells can incorporate themselves into the implanted scaffold and form vascular self-assembly structures in vivo. As previously discussed, using co-culture system can decrease apoptosis and increase implanted cell survival [110]. In vivo studies illustrate that the process of self-assembled of ECs occurs when HUVECs co-cultured with mouse mesenchymal precursors in collagen/fibronectin gel were transplanted in SCID mice. HUVECs self-assembled into micro-vessels, integrated into the host vasculature, could become perfused, and were stable and functional for one year [92]. Different cell sources that can be used for this purpose including iPSC-derived ECs in co-culture with MSCs, and smooth muscle cells or pericytes. It is important to note that perfusion of generated blood vessel is essential for long-term survival and tissue function. One strategy to generate perfusable blood vessels is to culture the constructs with specific cell types a few days prior to implantation, allowing for the pre-assembly of microvascular-like network. This method was successfully used in forming vascularized skeletal muscle fiber constructs in SCID mice using endothelial cells, mouse myoblasts, and mouse embryonic fibroblasts in biodegradable polylactic-acid and polylactic-glycolic scaffold (1:1) which demonstrated sufficient differentiation and elongation of cells after 2 weeks with improved vascularisation and survival [111]. Properly engineered microvasculature would greatly benefit the field of stem cell-derived organoids with the size > 6mm which requires a properly performed perfusion system to prevent the necrosis organoid development [112]. Co-culturing of human iPSC-derived hepatic cells with mesenchymal stem cells and HUVECs resulted in liver buds and could be perfused by host vasculature. Enhanced vascular density was observed when transplanted into the SCID mice [113]. Although, current engineered micro-vasculatures demonstrate perfusion by utilizing collections of cells in vivo, they are not yet efficient enough to reproduce the full function of the whole organ such as liver. Further study is required to expand the mass of cells that can be supported by engineered microvasculature. One key limitation in almost all gel-based endothelial microvascular self-assembly is that there is limited precision over the 3D orientations of the vessels formed. To address this, native organ scaffold and novel microfabrication technologies have been introduced which will be further discussed. 

### 4.3. Endothelialization of Decellularized Organs

The decellularization method is one of the most promising techniques for tissue regeneration in which the extracellular matrix (ECM) is isolated from native cells to produce a natural scaffold. Decellularized ECM maintains its mechanical, structural, and biochemical cues which can then be recellularized to produce a functional tissue mimicking the natural organization of specific tissue. Among different decellularization methods, the method that does not cause disruption of vascular integrity is the most suitable for cellular repopulation. Furthermore, proper endothelial repopulation of acellular scaffold is crucial to prevent microvascular coagulation stimulated by the collagenous matrix. This approach is one of the promising methods currently to generate robust perfusable microvasculature, though new microfabrication approaches are quickly developing [114,115].

### 4.4. Micropatterning Approaches to Microvessel Creation

Recently, the use of microfabrication techniques to produce controlled and reproducible patterns of vasculature have been widely investigated. Although these techniques are not ready for clinical applications, they are crucial developments to shape future directions. Durable engineered vasculature has important features including high mechanical properties to prevent rupture and accommodate flow with highly pulsatile pressure, low thrombogenicity, and high regenerative integration with the host tissue [105,116].

Recent advances in microfluidics and 3D bioprinting have been used to generate vascular constructs in tissue engineering applications. Microfluidic applications are divided into three different categories including sacrificial molding, micropatterning using soft lithography, and in vitro self-assembled endothelial cells [117]. All of these techniques are not clinically applicable in their present state. Sacrificial 3D printing to generate a vascular fabrication platform is another method in which a 3D architecture is first printed with carbohydrate glass materials, hardens, and then is uniformly embedded in a soluble ECM which crosslinks around the print. The chamber is then flushed out with water to form a hollow. ECs can then be seeded on the channel wall, forming the vascular bed [118]. With more recent advances, Kolesky and his colleagues [119] have been successful in generating an interconnected vascular network with perfusion capability showing that continuous perfusion of growth factors through the channel covered with HUVECs could successfully differentiate stem cells into osteogenic lineage. The important advantage of this technique is that the size and scale of the 3D construct can be sufficiently controlled. Another approach to pattern vascular structure is to use lithography where the 3D structure is fabricated additively. First, photolithography is used to fabricate each layer separately, and then all layers are chemically bonded using UV crosslinking or mechanically bonded [120,121,122]. This technique can produce microfluidic channels using a biodegradable polymer that branch out from an inlet channel and merge into an outlet channel like the vasculature system in vivo. Photo-crosslinking was used to fabricate these structures layer-by-layer, which were then fixed together to create hollow channels where EC can flow and eventually attach [28].

A final approach is to utilise endothelial cell ability to self-assemble into microvascular networks by applying interstitial flow to extracellular matrix-containing combinations of endothelial cells and mural cells within microfluidic chambers. These small-scale constructs (<1 mm^3^) are great platforms for disease modeling and drug testing. However, it is unknown if these constructs can further enhance stabilization and function of microvessels after in vivo transplantation. 

### 4.5. Laser-Degradation

Laser-based degradation of hydrogels, biomaterials, and other soft interfaces have recently garnered attention as a technique for obtaining high-resolution control over user-defined architectures within 3D tissue constructs and acellular platforms. Another method to generate a vascular network is to selectively degrade regions within a bulk crosslinked ECM. In this technique, a biomaterial is first crosslinked into a hydrogel, and then a laser is used to degrade the synthetic material locally. This technique was first used to fabricate channels with a diameter as small as 3 μm, though it was difficult to flow HUVECs through these channels without clogging [123]. Despite the very recent application of laser-based degradation within hydrogels, the continued improvement in laser and optical systems in combination with other larger-scale fabrication techniques and wide adoption for biofabrication will ultimately provide significant advancements in the field of cell and tissue engineering and regenerative medicine [124].

## 5. Conclusions

The proper formation of blood vessels is critical for the generation and continued function of tissues and organs during embryonic development and throughout life. Vascularization is the key challenge that hinders the clinical application of tissue-engineered products and, thus, the greatest obstacle to successful transplantation. To date, various strategies have been developed to generate vascular constructs at clinically relevant scales. However, the generation of vascular networks that can mimic the complexity, ultrastructure, geometry, and biochemical cues remains a challenge. Moreover, timely-mannered integration of engineered constructs after implantation is critical for optimum functionality of the implanted construct. To promote this, novel approaches have cultivated prevascularized constructs that can rapidly inoculate the implanted vascular construct to the host tissue, enhancing the survival of implanted constructs. 

Further, advanced imaging techniques enable the viewing and monitoring of the detailed structures in the integrated constructs over time. This derives from the current capacity of some optical imaging methods in clinical or preclinical research for micro-structural imaging or functional imaging [125,126,127,128,129,130,131,132,133] as well as in imaging nano-particles [9,134].

However, some of the drawbacks of these novel approaches are also important to be taken into consideration including tumourigenity, cell migration, graft rejection, immunogenity, and complexity of the host immune response [135]. The immunotolerant potential of implanted structures must be widely investigated. For example, fast leukocyte involvement into decellularized scaffold may prevent proper recellularization of autologous implants. The ability to modulate immune acceptance of cell-based implants remains a crucial barrier for the field. Another barrier that may inhibit successful implantation is the choice of biomaterials used as scaffold [136]. Effective specifications for the biomaterials have not been well-articulated. Conventional scaffold, especially when based on synthetic biodegradable polymers, is mechanistically inappropriate and the requirements for biodegradability and prior FDA approval should be fully addressed for use in medical devices [137]. At this time, none of the discussed approaches have the potential to be clinically applied. Thus, further improvement is needed to create perusable blood vessels which consist of multi-material and multicellular depositions, and that can closely mimic the physiological and mechanical properties of angiogenic systems in vivo.

## Figures and Tables

**Table 1:** Summary of four different anti-vascular endothelial growth factor (VEGF) drugs used in the treatment of diabetic retinopathy.

Name	Trade-Name	Description	Clinical Trials
**Pegaptanib**	Macugen—Eyetech New York	High affinity to the heparin binding site of VEGF-A isoforms.	FDA-approved for AMD but because of disappointment visual results, only used sparingly.
**Ranibizumab**	Lucentis—Genentech, S. Sam Francisco	Recombinant humanised anti-body fragment (Fab) that binds all isoforms of VEGF.	FDA-approved for AMD, macular edema & DME.
**Bevacizumab**	Avastin—Genentech	Recombinant full-length humanised monoclonal anti-body that also binds all VEGF isoforms.	FDA-approved for rectal carcinoma, ovarian carcinoma, glioblastoma but is off set for use in ocular diseases (AMD, DME & vein occlusion).
**Aflibercept**	Elyea—Regeneron, Tarrytown, NY	Recombinant fusion protein with native VEGFR ligand-binding sequences attached to the Fc segment of human IgG1. Binds all isoforms of VEGF-A, VEGF-B and placental growth factor.	FDA-approved for AMD & macular edema and the systemic formulation Zaltrap for colorectal carcinoma.

## References

[1] Nentwich M.M., Ulbig M.W. (2015). Diabetic retinopathy-ocular complications of diabetes mellitus. World J. Diabetes.

[2] Willard A.L., Herman I.M. (2012). Vascular complications and diabetes: Current therapies and future challenges. J. Ophthalmol..

[3] Stitt A.W., O’Neill C.L., O’Doherty M.T., Archer D.B., Gardiner T.A., Medina R.J. (2011). Vascular stem cells and ischaemic retinopathies. Prog. Retin. Eye Res..

[4] Chistiakov D.A. (2011). Diabetic retinopathy: Pathogenic mechanisms and current treatments. Diabetes Metab. Syndr..

[5] Wang Y., Yin P., Bian G.L., Huang H.Y., Shen H., Yang J.J., Yang Z.Y., Shen Z.Y. (2017). The combination of stem cells and tissue engineering: An advanced strategy for blood vessels regeneration and vascular disease treatment. Stem Cell Res. Ther..

[6] Rivera J.C., Dabouz R., Noueihed B., Omri S., Tahiri H., Chemtob S. (2017). Ischemic Retinopathies: Oxidative Stress and Inflammation. Oxid. Med. Cell. Longev..

[7] Prisco D., Marcucci R. (2002). Retinal vein thrombosis: Risk factors, pathogenesis and therapeutic approach. Pathophysiol. Haemost. Thromb..

[8] Durham J.T., Herman I.M. (2011). Microvascular modifications in diabetic retinopathy. Curr. Diab. Rep..

[9] Jalilian E., Xu Q., Horton L., Fotouhi A., Reddy S., Manwar R., Daveluy S., Mehregan D., Gelovani J., Avanaki K. (2020). Contrast-enhanced optical coherence tomography for melanoma detection: An in vitro study. J. Biophotonics.

[10] Ikram M.K., Ong Y.T., Cheung C.Y., Wong T.Y. (2013). Retinal vascular caliber measurements: Clinical significance, current knowledge and future perspectives. Ophthalmologica.

[11] Giuliari G.P. (2012). Diabetic retinopathy: Current and new treatment options. Curr. Diabetes Rev..

[12] Manousaridis K., Talks J. (2012). Macular ischaemia: A contraindication for anti-VEGF treatment in retinal vascular disease?. Br. J. Ophthalmol..

[13] Park Y.G., Roh Y.J. (2016). New Diagnostic and Therapeutic Approaches for Preventing the Progression of Diabetic Retinopathy. J. Diabetes Res..

[14] Duh E.J., Sun J.K., Stitt A.W. (2017). Diabetic retinopathy: Current understanding, mechanisms, and treatment strategies. JCI Insight.

[15] Wang X., Wang G., Wang Y. (2009). Intravitreous vascular endothelial growth factor and hypoxia-inducible factor 1a in patients with proliferative diabetic retinopathy. Am. J. Ophthalmol..

[16] Behl T., Kotwani A. (2015). Exploring the various aspects of the pathological role of vascular endothelial growth factor (VEGF) in diabetic retinopathy. Pharmacol. Res..

[17] Gupta N., Mansoor S., Sharma A., Sapkal A., Sheth J., Falatoonzadeh P., Kuppermann B., Kenney M. (2013). Diabetic retinopathy and VEGF. Open Ophthalmol. J..

[18] Virgili G., Parravano M., Menchini F., Evans J.R. (2014). Anti-vascular endothelial growth factor for diabetic macular oedema. Cochrane Database Syst. Rev..

[19] Ishida S., Usui T., Yamashiro K., Kaji Y., Ahmed E., Carrasquillo K.G., Amano S., Hida T., Oguchi Y., Adamis A.P. (2003). VEGF164 is proinflammatory in the diabetic retina. Investig. Ophthalmol. Vis. Sci..

[20] Cabral T., Mello L.G.M., Lima L.H., Polido J., Regatieri C.V., Belfort R., Mahajan V.B. (2017). Retinal and choroidal angiogenesis: A review of new targets. Int. J. Retina Vitr..

[21] Day S., Acquah K., Mruthyunjaya P., Grossman D.S., Lee P.P., Sloan F.A. (2011). Ocular complications after anti-vascular endothelial growth factor therapy in Medicare patients with age-related macular degeneration. Am. J. Ophthalmol..

[22] Ciulla T.A., Huang F., Westby K., Williams D.F., Zaveri S., Patel S.C. (2018). Real-world Outcomes of Anti-Vascular Endothelial Growth Factor Therapy in Neovascular Age-Related Macular Degeneration in the United States. Ophthalmol. Retina.

[23] Ciulla T.A., Hussain R.M., Pollack J.S., Williams D.F. (2020). Visual Acuity Outcomes and Anti-Vascular Endothelial Growth Factor Therapy Intensity in Neovascular Age-Related Macular Degeneration Patients: A Real-World Analysis of 49 485 Eyes. Ophthalmol. Retina.

[24] Foxton R.H., Finkelstein A., Vijay S., Dahlmann-Noor A., Khaw P.T., Morgan J.E., Shima D.T., Ng Y.S. (2013). VEGF-A is necessary and sufficient for retinal neuroprotection in models of experimental glaucoma. Am. J. Pathol..

[25] Nishijima K., Ng Y.S., Zhong L., Bradley J., Schubert W., Jo N., Akita J., Samuelsson S.J., Robinson G.S., Adamis A.P. (2007). Vascular endothelial growth factor-A is a survival factor for retinal neurons and a critical neuroprotectant during the adaptive response to ischemic injury. Am. J. Pathol..

[26] Mackenzie F., Ruhrberg C. (2012). Diverse roles for VEGF-A in the nervous system. Development.

[27] Min S., Ko I.K., Yoo J.J. (2019). State-of-the-Art Strategies for the Vascularization of Three-Dimensional Engineered Organs. Vasc. Spec. Int..

[28] Song H.G., Rumma R.T., Ozaki C.K., Edelman E.R., Chen C.S. (2018). Vascular Tissue Engineering: Progress, Challenges, and Clinical Promise. Cell Stem Cell.

[29] Sarker M.D., Naghieh S., Sharma N.K., Chen X. (2018). 3D biofabrication of vascular networks for tissue regeneration: A report on recent advances. J. Pharm. Anal..

[30] Ribatti D., Nico B., Crivellato E. (2015). The development of the vascular system: A historical overview. Methods Mol. Biol..

[31] Bautch V.L., Caron K.M. (2015). Blood and lymphatic vessel formation. Cold Spring Harb. Perspect. Biol..

[32] Patan S. (2004). Vasculogenesis and angiogenesis. Cancer Treat Res..

[33] Carmeliet P. (2005). Angiogenesis in life, disease and medicine. Nature.

[34] Jalilian E., Powner M.B., Fruttiger M.J.I.O., Science V. (2014). Tip cell signalling in Endothelial Cells. Investig. Ophthalmol. Vis. Sci..

[35] Marcelo K.L., Goldie L.C., Hirschi K.K. (2013). Regulation of endothelial cell differentiation and specification. Circ. Res..

[36] Stratman A.N., Pezoa S.A., Farrelly O.M., Castranova D., Dye L.E., Butler M.G., Sidik H., Talbot W.S., Weinstein B.M. (2017). Interactions between mural cells and endothelial cells stabilize the developing zebrafish dorsal aorta. Development.

[37] Pardali E., Goumans M.J., ten Dijke P. (2010). Signaling by members of the TGF-beta family in vascular morphogenesis and disease. Trends Cell Biol..

[38] Kume T. (2009). Novel insights into the differential functions of Notch ligands in vascular formation. J. Angiogenes Res..

[39] Mack J.J., Iruela-Arispe M.L. (2018). NOTCH regulation of the endothelial cell phenotype. Curr. Opin. Hematol..

[40] Kale S., Hanai J., Chan B., Karihaloo A., Grotendorst G., Cantley L., Sukhatme V.P. (2005). Microarray analysis of in vitro pericyte differentiation reveals an angiogenic program of gene expression. FASEB J..

[41] Au P., Daheron L.M., Duda D.G., Cohen K.S., Tyrrell J.A., Lanning R.M., Fukumura D., Scadden D.T., Jain R.K. (2008). Differential in vivo potential of endothelial progenitor cells from human umbilical cord blood and adult peripheral blood to form functional long-lasting vessels. Blood.

[42] Melero-Martin J.M., Khan Z.A., Picard A., Wu X., Paruchuri S., Bischoff J. (2007). In vivo vasculogenic potential of human blood-derived endothelial progenitor cells. Blood.

[43] Foubert P., Matrone G., Souttou B., Lere-Dean C., Barateau V., Plouet J., Le Ricousse-Roussanne S., Levy B.I., Silvestre J.S., Tobelem G. (2008). Coadministration of endothelial and smooth muscle progenitor cells enhances the efficiency of proangiogenic cell-based therapy. Circ. Res..

[44] Kawamoto A., Asahara T., Losordo D.W. (2002). Transplantation of endothelial progenitor cells for therapeutic neovascularization. Cardiovasc. Radiat. Med..

[45] Ribatti D. (2007). The discovery of endothelial progenitor cells. An historical review. Leuk. Res..

[46] Asahara T., Kawamoto A., Masuda H. (2011). Concise review: Circulating endothelial progenitor cells for vascular medicine. Stem Cells.

[47] Harris D.T., Rogers I. (2007). Umbilical cord blood: A unique source of pluripotent stem cells for regenerative medicine. Curr. Stem Cell Res. Ther..

[48] Peichev M., Naiyer A.J., Pereira D., Zhu Z., Lane W.J., Williams M., Oz M.C., Hicklin D.J., Witte L., Moore M.A. (2000). Expression of VEGFR-2 and AC133 by circulating human CD34(+) cells identifies a population of functional endothelial precursors. Blood.

[49] Asahara T., Murohara T., Sullivan A., Silver M., van der Zee R., Li T., Witzenbichler B., Schatteman G., Isner J.M. (1997). Isolation of putative progenitor endothelial cells for angiogenesis. Science.

[50] Yang J., Ii M., Kamei N., Alev C., Kwon S.M., Kawamoto A., Akimaru H., Masuda H., Sawa Y., Asahara T. (2011). CD34+ cells represent highly functional endothelial progenitor cells in murine bone marrow. PLoS ONE.

[51] Miller-Kasprzak E., Jagodzinski P.P. (2007). Endothelial progenitor cells as a new agent contributing to vascular repair. Arch. Immunol. Ther. Exp. (Warsz.).

[52] Sharpe E.E., Teleron A.A., Li B., Price J., Sands M.S., Alford K., Young P.P. (2006). The origin and in vivo significance of murine and human culture-expanded endothelial progenitor cells. Am. J. Pathol..

[53] Timmermans F., Plum J., Yoder M.C., Ingram D.A., Vandekerckhove B., Case J. (2009). Endothelial progenitor cells: Identity defined?. J. Cell Mol. Med..

[54] Jalilian E., Sim D.A., Powner M.B., Coffey P., Fruttiger M.J.I.O., Science V. (2015). Isolation Endothelial Progenitor Cells (EPCs) from Induced Pluripotent Stem Cells (iPSC) cells. Investig. Ophthalmol. Vis. Sci.

[55] Huang N.F., Niiyama H., Peter C., De A., Natkunam Y., Fleissner F., Li Z., Rollins M.D., Wu J.C., Gambhir S.S. (2010). Embryonic stem cell-derived endothelial cells engraft into the ischemic hindlimb and restore perfusion. Arterioscler. Thromb. Vasc. Biol..

[56] Koizumi K., Tsutsumi Y., Kamada H., Yoshioka Y., Watanabe M., Yamamoto Y., Okamoto T., Mukai Y., Nakagawa S., Tani Y. (2003). Incorporation of adult organ-derived endothelial cells into tumor blood vessel. Biochem. Biophys. Res. Commun..

[57] Kawamoto A., Gwon H.C., Iwaguro H., Yamaguchi J.I., Uchida S., Masuda H., Silver M., Ma H., Kearney M., Isner J.M. (2001). Therapeutic potential of ex vivo expanded endothelial progenitor cells for myocardial ischemia. Circulation.

[58] Shaw L.C., Neu M.B., Grant M.B. (2011). Cell-based therapies for diabetic retinopathy. Curr. Diab. Rep..

[59] Ritter M.R., Banin E., Moreno S.K., Aguilar E., Dorrell M.I., Friedlander M. (2006). Myeloid progenitors differentiate into microglia and promote vascular repair in a model of ischemic retinopathy. J. Clin. Investig.

[60] Rajashekhar G., Ramadan A., Abburi C., Callaghan B., Traktuev D.O., Evans-Molina C., Maturi R., Harris A., Kern T.S., March K.L. (2014). Regenerative therapeutic potential of adipose stromal cells in early stage diabetic retinopathy. PLoS ONE.

[61] Holan V., Hermankova B., Kossl J. (2017). Perspectives of Stem Cell-Based Therapy for Age-Related Retinal Degenerative Diseases. Cell Transpl..

[62] Sietsema W.K., Kawamoto A., Takagi H., Losordo D.W. (2019). Autologous CD34+ Cell Therapy for Ischemic Tissue Repair. Circ. J..

[63] Urbich C., Dimmeler S. (2004). Endothelial progenitor cells: Characterization and role in vascular biology. Circ. Res..

[64] Balaji S., King A., Crombleholme T.M., Keswani S.G. (2013). The Role of Endothelial Progenitor Cells in Postnatal Vasculogenesis: Implications for Therapeutic Neovascularization and Wound Healing. Adv. Wound Care (New Rochelle).

[65] Palis J., Robertson S., Kennedy M., Wall C., Keller G. (1999). Development of erythroid and myeloid progenitors in the yolk sac and embryo proper of the mouse. Development.

[66] Medina R.J., O’Neill C.L., Humphreys M.W., Gardiner T.A., Stitt A.W. (2010). Outgrowth endothelial cells: Characterization and their potential for reversing ischemic retinopathy. Investig. Ophthalmol. Vis. Sci..

[67] Prasain N., Lee M.R., Vemula S., Meador J.L., Yoshimoto M., Ferkowicz M.J., Fett A., Gupta M., Rapp B.M., Saadatzadeh M.R. (2014). Differentiation of human pluripotent stem cells to cells similar to cord-blood endothelial colony-forming cells. Nat. Biotechnol..

[68] Yoder M.C., Mead L.E., Prater D., Krier T.R., Mroueh K.N., Li F., Krasich R., Temm C.J., Prchal J.T., Ingram D.A. (2007). Redefining endothelial progenitor cells via clonal analysis and hematopoietic stem/progenitor cell principals. Blood.

[69] Peters E.B., Christoforou N., Leong K.W., Truskey G.A., West J.L. (2016). Poly(ethylene glycol) Hydrogel Scaffolds Containing Cell-Adhesive and Protease-Sensitive Peptides Support Microvessel Formation by Endothelial Progenitor Cells. Cell Mol. Bioeng..

[70] Melero-Martin J.M., De Obaldia M.E., Kang S.Y., Khan Z.A., Yuan L., Oettgen P., Bischoff J. (2008). Engineering robust and functional vascular networks in vivo with human adult and cord blood-derived progenitor cells. Circ. Res..

[71] Melchiorri A.J., Bracaglia L.G., Kimerer L.K., Hibino N., Fisher J.P. (2016). In Vitro Endothelialization of Biodegradable Vascular Grafts Via Endothelial Progenitor Cell Seeding and Maturation in a Tubular Perfusion System Bioreactor. Tissue Eng. Part C Methods.

[72] Lu C.C., Brennan J., Robertson E.J. (2001). From fertilization to gastrulation: Axis formation in the mouse embryo. Curr. Opin. Genet. Dev..

[73] Watt S.M., Contreras M. (2005). Stem cell medicine: Umbilical cord blood and its stem cell potential. Semin. Fetal Neonatal Med..

[74] Volz K.S., Miljan E., Khoo A., Cooke J.P. (2012). Development of pluripotent stem cells for vascular therapy. Vascul. Pharmacol..

[75] Zovein A.C., Hofmann J.J., Lynch M., French W.J., Turlo K.A., Yang Y., Becker M.S., Zanetta L., Dejana E., Gasson J.C. (2008). Fate tracing reveals the endothelial origin of hematopoietic stem cells. Cell Stem Cell.

[76] Levenberg S., Ferreira L.S., Chen-Konak L., Kraehenbuehl T.P., Langer R. (2010). Isolation, differentiation and characterization of vascular cells derived from human embryonic stem cells. Nat. Protoc..

[77] Takahashi K., Yamanaka S. (2006). Induction of pluripotent stem cells from mouse embryonic and adult fibroblast cultures by defined factors. Cell.

[78] Caspi O., Huber I., Kehat I., Habib M., Arbel G., Gepstein A., Yankelson L., Aronson D., Beyar R., Gepstein L. (2007). Transplantation of human embryonic stem cell-derived cardiomyocytes improves myocardial performance in infarcted rat hearts. J. Am. Coll. Cardiol..

[79] Nelson T.J., Martinez-Fernandez A., Yamada S., Perez-Terzic C., Ikeda Y., Terzic A. (2009). Repair of acute myocardial infarction by human stemness factors induced pluripotent stem cells. Circulation.

[80] Saric T., Frenzel L.P., Hescheler J. (2008). Immunological barriers to embryonic stem cell-derived therapies. Cells Tissues Organs.

[81] Blum B., Benvenisty N. (2008). The tumorigenicity of human embryonic stem cells. Adv. Cancer Res..

[82] Park I.H., Arora N., Huo H., Maherali N., Ahfeldt T., Shimamura A., Lensch M.W., Cowan C., Hochedlinger K., Daley G.Q. (2008). Disease-specific induced pluripotent stem cells. Cell.

[83] Hanna J., Wernig M., Markoulaki S., Sun C.W., Meissner A., Cassady J.P., Beard C., Brambrink T., Wu L.C., Townes T.M. (2007). Treatment of sickle cell anemia mouse model with iPS cells generated from autologous skin. Science.

[84] Narazaki G., Uosaki H., Teranishi M., Okita K., Kim B., Matsuoka S., Yamanaka S., Yamashita J.K. (2008). Directed and systematic differentiation of cardiovascular cells from mouse induced pluripotent stem cells. Circulation.

[85] Mauritz C., Schwanke K., Reppel M., Neef S., Katsirntaki K., Maier L.S., Nguemo F., Menke S., Haustein M., Hescheler J. (2008). Generation of functional murine cardiac myocytes from induced pluripotent stem cells. Circulation.

[86] Li Z., Wu J.C., Sheikh A.Y., Kraft D., Cao F., Xie X., Patel M., Gambhir S.S., Robbins R.C., Cooke J.P. (2007). Differentiation, survival, and function of embryonic stem cell derived endothelial cells for ischemic heart disease. Circulation.

[87] Yamahara K., Sone M., Itoh H., Yamashita J.K., Yurugi-Kobayashi T., Homma K., Chao T.H., Miyashita K., Park K., Oyamada N. (2008). Augmentation of neovascularization [corrected] in hindlimb ischemia by combined transplantation of human embryonic stem cells-derived endothelial and mural cells. PLoS ONE.

[88] Okita K., Hong H., Takahashi K., Yamanaka S. (2010). Generation of mouse-induced pluripotent stem cells with plasmid vectors. Nat. Protoc..

[89] Sommer C.A., Stadtfeld M., Murphy G.J., Hochedlinger K., Kotton D.N., Mostoslavsky G. (2009). Induced pluripotent stem cell generation using a single lentiviral stem cell cassette. Stem Cells.

[90] Jalilian E., Raimes W. (2020). Transcriptional profiling reveals fundamental differences in iPS-derived progenitors of endothelial cells (PECs) versus adult circulating EPCs. arXiv.

[91] Jalilian E. (2017). Characterisation of Progenitors of Endothelial Cells (PECs). Ph.D. Thesis.

[92] Koike N., Fukumura D., Gralla O., Au P., Schechner J.S., Jain R.K. (2004). Tissue engineering: Creation of long-lasting blood vessels. Nature.

[93] Cao Y., Sun Z., Liao L., Meng Y., Han Q., Zhao R.C. (2005). Human adipose tissue-derived stem cells differentiate into endothelial cells in vitro and improve postnatal neovascularization in vivo. Biochem. Biophys. Res. Commun..

[94] Xu Y., Meng H., Li C., Hao M., Wang Y., Yu Z., Li Q., Han J., Zhai Q., Qiu L. (2010). Umbilical cord-derived mesenchymal stem cells isolated by a novel explantation technique can differentiate into functional endothelial cells and promote revascularization. Stem Cells Dev..

[95] Narita Y., Yamawaki A., Kagami H., Ueda M., Ueda Y. (2008). Effects of transforming growth factor-beta 1 and ascorbic acid on differentiation of human bone-marrow-derived mesenchymal stem cells into smooth muscle cell lineage. Cell Tissue Res..

[96] Yang G., Mahadik B., Choi J.Y., Fisher J.P. (2020). Vascularization in tissue engineering: Fundamentals and state-of-art. Prog. Biomed. Eng..

[97] Rodriguez L.G., Wu X., Guan J.L. (2005). Wound-healing assay. Methods Mol. Biol..

[98] Baker M., Robinson S.D., Lechertier T., Barber P.R., Tavora B., D’Amico G., Jones D.T., Vojnovic B., Hodivala-Dilke K. (2011). Use of the mouse aortic ring assay to study angiogenesis. Nat. Protoc..

[99] Bellacen K., Lewis E.C. (2009). Aortic ring assay. J. Vis. Exp..

[100] Koh W., Stratman A.N., Sacharidou A., Davis G.E. (2008). In vitro three dimensional collagen matrix models of endothelial lumen formation during vasculogenesis and angiogenesis. Methods Enzymol..

[101] Yang S., Graham J., Kahn J.W., Schwartz E.A., Gerritsen M.E. (1999). Functional roles for PECAM-1 (CD31) and VE-cadherin (CD144) in tube assembly and lumen formation in three-dimensional collagen gels. Am. J. Pathol..

[102] Nakatsu M.N., Davis J., Hughes C.C. (2007). Optimised fibrin gel bead assay for the study of angiogenesis. J. Vis. Exp..

[103] Jolicoeur C., Cayouette M. (2014). Retinal Explant Culture. Bio-Protocol..

[104] Kechad A., Jolicoeur C., Tufford A., Mattar P., Chow R.W.Y., Harris W.A., Cayouette M. (2012). Numb is required for the production of terminal asymmetric cell divisions in the developing mouse retina. J. Neurosci..

[105] Chang W.G., Niklason L.E. (2017). A short discourse on vascular tissue engineering. NPJ Regen. Med..

[106] Chu H., Wang Y. (2012). Therapeutic angiogenesis: Controlled delivery of angiogenic factors. Ther. Deliv..

[107] Hedman M., Hartikainen J., Yla-Herttuala S. (2011). Progress and prospects: Hurdles to cardiovascular gene therapy clinical trials. Gene Ther..

[108] Losordo D.W., Dimmeler S. (2004). Therapeutic angiogenesis and vasculogenesis for ischemic disease: Part II: Cell-based therapies. Circulation.

[109] Leeper N.J., Hunter A.L., Cooke J.P. (2010). Stem cell therapy for vascular regeneration: Adult, embryonic, and induced pluripotent stem cells. Circulation.

[110] Ilan N., Mahooti S., Madri J.A. (1998). Distinct signal transduction pathways are utilized during the tube formation and survival phases of in vitro angiogenesis. J. Cell Sci..

[111] Levenberg S., Rouwkema J., Macdonald M., Garfein E.S., Kohane D.S., Darland D.C., Marini R., van Blitterswijk C.A., Mulligan R.C., D’Amore P.A. (2005). Engineering vascularized skeletal muscle tissue. Nat. Biotechnol..

[112] Takebe T., Zhang R.R., Koike H., Kimura M., Yoshizawa E., Enomura M., Koike N., Sekine K., Taniguchi H. (2014). Generation of a vascularized and functional human liver from an iPSC-derived organ bud transplant. Nat. Protoc..

[113] Takebe T., Sekine K., Enomura M., Koike H., Kimura M., Ogaeri T., Zhang R.R., Ueno Y., Zheng Y.W., Koike N. (2013). Vascularized and functional human liver from an iPSC-derived organ bud transplant. Nature.

[114] Wells W.A. (1998). Could mitochondria be the key?. Chem. Biol..

[115] Kolster M., Wilhelmi M., Schrimpf C., Hilfiker A., Haverich A., Aper T. (2017). Outgrowing endothelial and smooth muscle cells for tissue engineering approaches. J. Tissue Eng..

[116] Saghazadeh S., Rinoldi C., Schot M., Kashaf S.S., Sharifi F., Jalilian E., Nuutila K., Giatsidis G., Mostafalu P., Derakhshandeh H. (2018). Drug delivery systems and materials for wound healing applications. Adv. Drug Deliv. Rev..

[117] Massa S., Sakr M.A., Seo J., Bandaru P., Arneri A., Bersini S., Zare-Eelanjegh E., Jalilian E., Cha B.H., Antona S. (2017). Bioprinted 3D vascularized tissue model for drug toxicity analysis. Biomicrofluidics.

[118] Miller J.S., Stevens K.R., Yang M.T., Baker B.M., Nguyen D.-H.T., Cohen D.M., Toro E., Chen A.A., Galie P.A., Yu X. (2012). Rapid casting of patterned vascular networks for perfusable engineered three-dimensional tissues. Nat. Mater..

[119] Kolesky D.B., Homan K.A., Skylar-Scott M.A., Lewis J.A. (2016). Three-dimensional bioprinting of thick vascularized tissues. Proc. Natl. Acad. Sci. USA.

[120] Zhang B., Montgomery M., Chamberlain M.D., Ogawa S., Korolj A., Pahnke A., Wells L.A., Masse S., Kim J., Reis L. (2016). Biodegradable scaffold with built-in vasculature for organ-on-a-chip engineering and direct surgical anastomosis. Nat. Mater..

[121] Morgan J.P., Delnero P.F., Zheng Y., Verbridge S.S., Chen J., Craven M., Choi N.W., Diaz-Santana A., Kermani P., Hempstead B. (2013). Formation of microvascular networks in vitro. Nat. Protoc..

[122] Zheng Y., Chen J., Craven M., Choi N.W., Totorica S., Diaz-Santana A., Kermani P., Hempstead B., Fischbach-Teschl C., Lopez J.A. (2012). In vitro microvessels for the study of angiogenesis and thrombosis. Proc. Natl. Acad. Sci. USA.

[123] Heintz K.A., Bregenzer M.E., Mantle J.L., Lee K.H., West J.L., Slater J.H. (2016). Fabrication of 3D Biomimetic Microfluidic Networks in Hydrogels. Adv. Healthc. Mater..

[124] Pradhan S., Keller K.A., Sperduto J.L., Slater J.H. (2017). Fundamentals of Laser-Based Hydrogel Degradation and Applications in Cell and Tissue Engineering. Adv. Healthc. Mater..

[125] Avanaki M.R.N., Hojjat A., Podoleanu A.G. (2009). Investigation of computer-based skin cancer detection using optical coherence tomography. J. Mod. Opt..

[126] Avanaki M.R.N., Hojjatoleslami A. (2013). Skin layer detection of optical coherence tomography images. Optik.

[127] Rajabi-Estarabadi A., Bittar J.M., Zheng C., Nascimento V., Camacho I., Feun L.G., Nasiriavanaki M., Kunz M., Nouri K. (2019). Optical coherence tomography imaging of melanoma skin cancer. Lasers Med. Sci..

[128] O’Leary S., Fotouhi A., Turk D., Sriranga P., Rajabi-Estarabadi A., Nouri K., Daveluy S., Mehregan D., Nasiriavanaki M. (2018). OCT image atlas of healthy skin on sun-exposed areas. Skin Res. Technol..

[129] Mohammadi-Nejad A.-R., Mahmoudzadeh M., Hassanpour M.S., Wallois F., Muzik O., Papadelis C., Hansen A., Soltanian-Zadeh H., Gelovani J., Nasiriavanaki M. (2018). Neonatal brain resting-state functional connectivity imaging modalities. Photoacoustics.

[130] Turani Z., Fatemizadeh E., Blumetti T., Daveluy S., Moraes A.F., Chen W., Mehregan D., Andersen P.E., Nasiriavanaki M. (2019). Optical Radiomic Signatures Derived from Optical Coherence Tomography Images Improve Identification of Melanoma. Cancer Res..

[131] Adabi S., Hosseinzadeh M., Noei S., Conforto S., Daveluy S., Clayton A., Mehregan D., Nasiriavanaki M. (2017). Universal in vivo Textural Model for Human Skin based on Optical Coherence Tomograms. Sci. Rep..

[132] Yao J., Xia J., Maslov K.I., Nasiriavanaki M., Tsytsarev V., Demchenko A.V., Wang L.V. (2013). Noninvasive photoacoustic computed tomography of mouse brain metabolism in vivo. NeuroImage.

[133] Nasiriavanaki M., Xia J., Wan H., Bauer A.Q., Culver J.P., Wang L.V. (2014). High-resolution photoacoustic tomography of resting-state functional connectivity in the mouse brain. Proc. Natl. Acad. Sci. USA.

[134] Xu Q., Jalilian E., Fakhoury J.W., Manwar R., Michniak-Kohn B., Elkin K.B., Avanaki K. (2020). Monitoring the topical delivery of ultrasmall gold nanoparticles using optical coherence tomography. Skin Res. Technol..

[135] Zakrzewski J.L., van den Brink M.R., Hubbell J.A. (2014). Overcoming immunological barriers in regenerative medicine. Nat. Biotechnol..

[136] Andorko J.I., Jewell C.M. (2017). Designing biomaterials with immunomodulatory properties for tissue engineering and regenerative medicine. Bioeng. Transl. Med..

[137] Williams D.F. (2019). Challenges with the Development of Biomaterials for Sustainable Tissue Engineering. Front. Bioeng. Biotechnol..

